# A case of walled‐off necrosis extending into the pelvic cavity successfully treated by endoscopic necrosectomy using a lumen‐apposing metal stent

**DOI:** 10.1002/deo2.120

**Published:** 2022-04-23

**Authors:** Makoto Morita, Tomoyuki Yokota, Ryo Yano, Michiko Amano, Hironori Ochi, Nobuaki Azemoto, Toshie Mashiba, Kouji Joko

**Affiliations:** ^1^ Center for Liver‐Biliary‐Pancreatic Diseases Matsuyama Red Cross Hospital Ehime Japan

**Keywords:** acute pancreatitis, endoscopic necrosectomy, endoscopic ultrasound‐guided pancreatic cyst drainage, lumen‐apposing metal stent, walled‐off necrosis

## Abstract

A 31‐year‐old man developed massive walled‐off necrosis extending into the pelvic cavity following severe acute alcoholic pancreatitis. Endoscopic ultrasound‐guided fistula drainage was performed using a lumen‐apposing metal stent, but this was insufficiently effective, and endoscopic necrosectomy was also performed, after which the patient improved. Percutaneous drainage and surgery are other options for the treatment of walled‐off necrosis extending into the pelvic cavity, but a valuable case in which the patient improved with endoscopic treatment alone is presented.

## INTRODUCTION

A lumen‐apposing metal stent (LAMS) is a metal stent used to create a fistula by grasping two separate lumina with two large flanges and bringing them together. Its large aperture diameter means that an upper gastrointestinal endoscope can be inserted into the stent lumen, making it extremely effective in necrosectomy to treat walled‐off necrosis (WON) The successful use of endoscopic necrosectomy using a LAMS to treat a patient with WON extending into the pelvic cavity following severe pancreatitis is described.

## CASE REPORT

**FIGURE 1:**
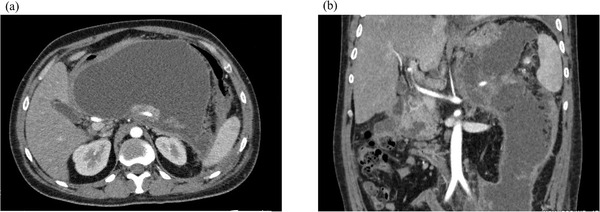
Abdominal contrast‐enhanced computed tomography (pre‐treatment). (a) Massive acute necrotic collection is evident in the upper abdomen. (b) The acute necrotic collection extends continuously from the upper abdomen into the pelvic cavity

**FIGURE 2:**
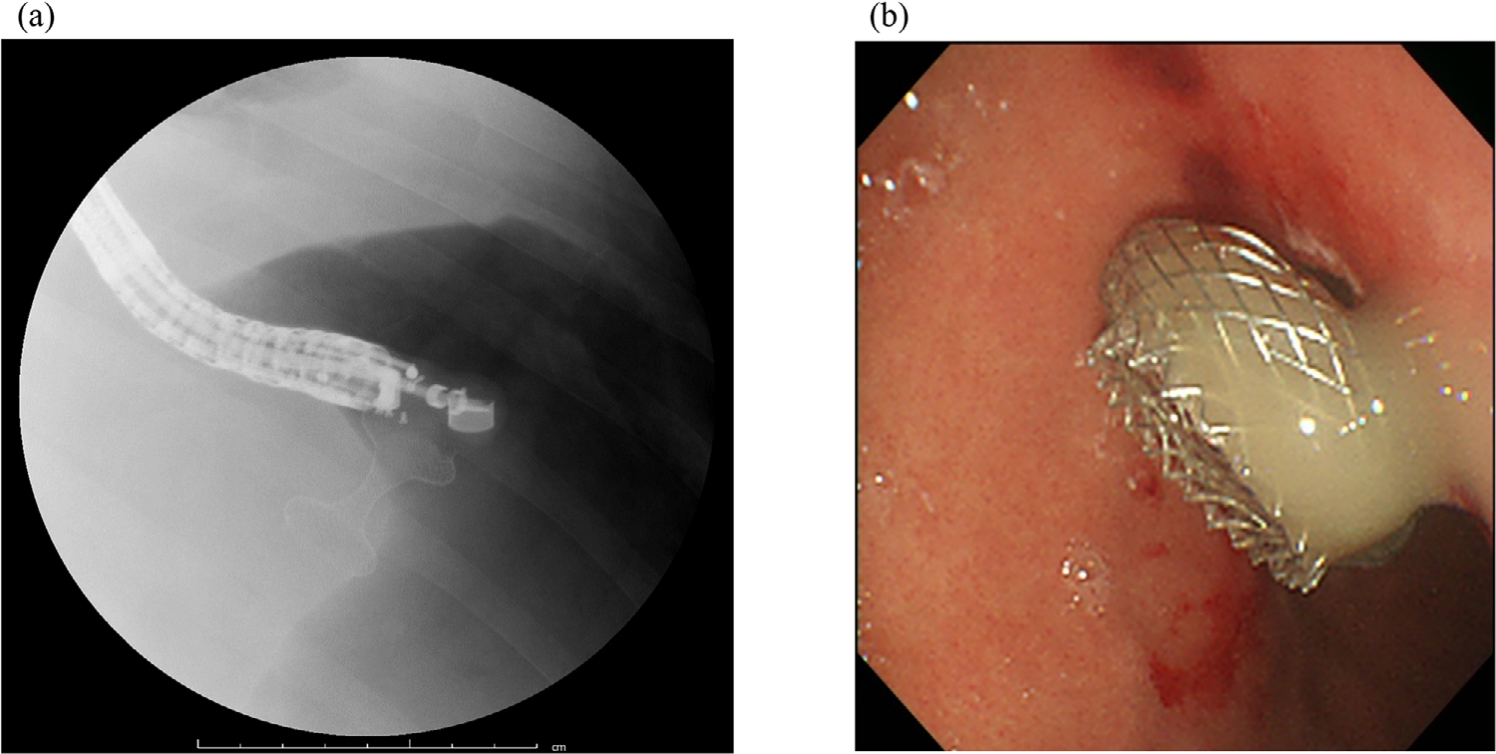
Fluoroscopy image and endoscopic view during lumen‐apposing metal stent placement. (a) The lumen‐apposing metal stent is inserted into the walled‐off necrosis, and the distal flange is opened, with the proximal flange opened inside the stomach. (b) Cloudy‐white pus can be seen to be draining from the lumen‐apposing metal stent placed from the posterior wall of the gastric corpus

**FIGURE 3:**
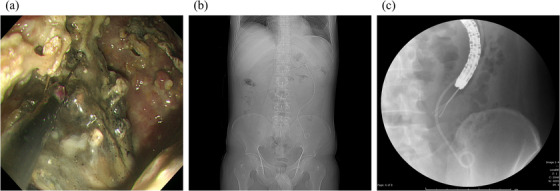
Endoscopic view and fluoroscopy image during necrosectomy. (a) Necrotic tissue within the walled‐off necrosis is removed with grasping forceps. (b) Abdominal X‐ray after insertion of the double pigtail plastic ureteral stent. (c) Endoscopic necrosectomy and drainage via a double pigtail plastic ureteral stent are conducted for the walled‐off necrosis in the pelvic cavity

**FIGURE 4:**
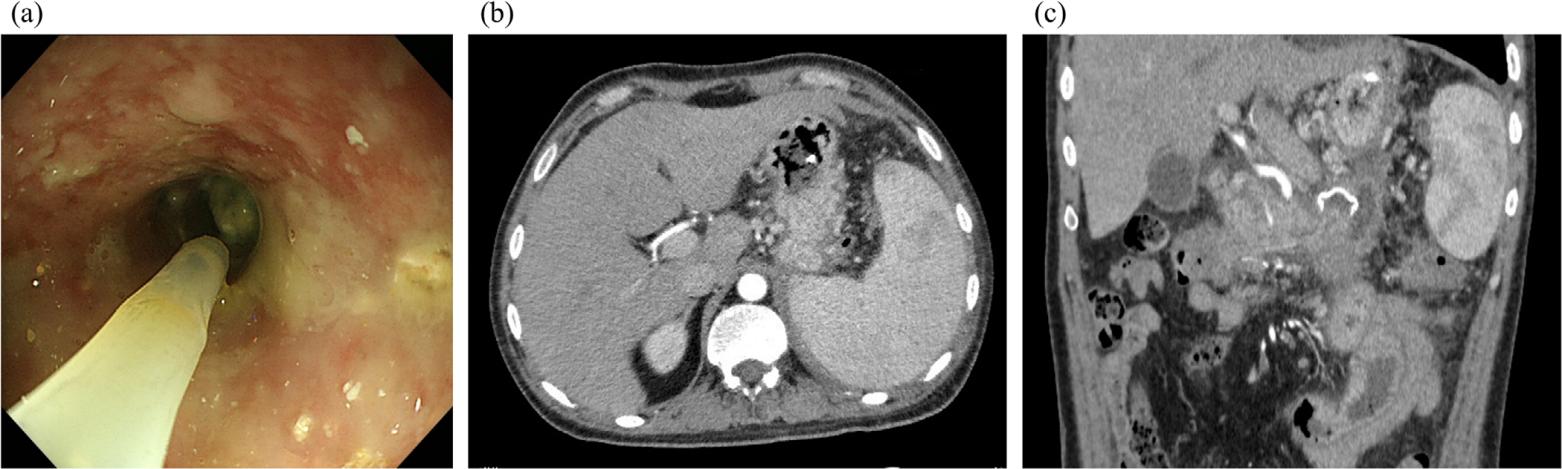
Endoscopic view and abdominal contrast‐enhanced computed tomography (post‐treatment). (a) The condition is greatly improved by endoscopic necrosectomy and drainage. (b, c) The walled‐off necrosis has almost completely disappeared

A 31‐year‐old man presented at another hospital in April 2020 with a chief complaint of abdominal pain. He had been drinking two glasses of whisky a week since the age of 25 years and had a history of acute alcoholic pancreatitis. Severe acute pancreatitis was diagnosed based on findings from blood tests and computed tomography (CT), and he was admitted to the hospital. On Day 4 his urine output decreased and abdominal pain worsened, blood tests showed markedly elevated lactate dehydrogenase and C‐reactive protein levels, and he was transferred to our hospital. On admission, he had a prognostic factor score of 3 (blood urea nitrogen, lactate dehydrogenase, and C‐reactive protein) according to the Japanese Criteria for Severity Assessment of Acute Pancreatitis of the Ministry of Health, Labour and Welfare, and CT demonstrated grade 3 severe acute pancreatitis. He was therefore started on high‐volume fluid infusion (4000 ml/day), intravenous antibiotic infusion (meropenem 3 g/day), and continuous intravenous nafamostat mesilate infusion (240 mg/day), and continuous intravenous buprenorphine hydrochloride infusion (2.4 mg/day). On Day 6, the abdominal pain improved, urine output increased, and C‐reactive protein also started to improve; therefore, he was started on an elemental diet. On Day 7, as his prognostic factor score improved to 2, he was started on a fat‐restricted diet. On Day 9, the abdominal pain relapsed, and he had continuous fever. Abdominal contrast‐enhanced CT showed an acute necrotic collection extending from the upper abdomen into the pelvic cavity (Figure 1a,b). This failed to improve with conservative therapy, and on Day 28, endoscopic ultrasound‐guided fistula drainage using a LAMS was performed (Figure 2a,b). The acute necrotic collection decreased in size, and the inflammatory response also started to improve, but from Day 32, the inflammatory response again increased, and CT showed WON formation from the left retroperitoneal space to the pelvic cavity. On Day 33, a direct‐view upper gastrointestinal endoscope was inserted via the LAMS, and the left retroperitoneal space was approached with an endoscopic retrograde cholangiopancreatography catheter and guidewire. Since it was possible to advance the guidewire into the pelvic cavity, a pigtail drainage tube was put in place and used as a transnasal external fistula. However, the patient's fever persisted, and only a small amount of fluid drained from the tube. It was decided that treatment by drainage alone was unlikely to be successful, and on Day 42, an initial necrosectomy was performed. As much as possible of the necrotic tissue around the pancreas was removed with grasping forceps and tripod forceps (Figure 3a), and since a biliary stent was not long enough to reach the WON in the pelvic cavity, it was decided to use a double pigtail plastic ureteral stent (Percuflex Plus Stent 7‐Fr × 30 cm; Boston Scientific, Natick, MA, USA). A guidewire (Hydra Jagwire Angled Tip Guidewire 0.035 inch × 260 cm; Boston Scientific) was inserted into the pelvic cavity, and the stent was put in place using a pusher catheter (GF stent set, 7‐Fr × 270 cm; GADELIUS, Tokyo, Japan) (Figure 3b). The use of the ureteral stent was approved by our ethics committee in advance. However, the patient's fever persisted, so an endoscope was inserted into the WON in the pelvic cavity while the site of stenosis within the WON was dilated with a balloon, and a necrosectomy of this area was also performed (Figure 3c). Necrosectomy was performed a total of five times until the WON from the left retroperitoneal space to the pelvic cavity had almost disappeared. Some WON remained on the anterior side of the head of the pancreas, but since it contained little necrotic tissue, and there was a risk that necrosectomy might damage the gastroduodenal artery, a double pigtail plastic biliary stent (Through and Pass Double Pigtail 7‐Fr × 18 cm; GADELIUS) was placed for drainage. Because the WON then almost disappeared (Figure 4a–c), on Day 74, the LAMS was removed while the plastic stent was left in place. The subsequent course was uneventful, and on Day 85, the patient was discharged.

## DISCUSSION

The value of endoscopic necrosectomy using LAMS for WON has been widely reported in recent years. Following endoscopic ultrasound‐guided drainage, necrosectomy can be started without having to wait for fistula formation by using LAMS, shortening the duration of treatment. Since the endoscope can be inserted and removed smoothly, necrotic tissue can be removed easily. Mukai et al. reported good results from the use of endoscopic ultrasound‐guided drainage and endoscopic necrosectomy via a LAMS, with a procedural success rate of 100% and response to treatment of 97.7%.^1^ Regarding complications, bleeding, perforation, stent deviation, and so forth have been reported.^2^ It has also been reported that stent deviation increases cumulatively with long‐term placement,^3^ and the official instruction manual recommends placement within 60 days. In the present case as well, the LAMS was removed 46 days after placement. After removal of the LAMS, plastic stents should be placed in the fistula as much as possible to prevent a recurrence. Especially in cases with widespread necrosis such as the present patient, disconnected pancreatic duct syndrome may occur, and it is said that transmural stents should be left in place for a long time.^4^

The multiple transluminal gateway technique (MTGT)^5^ and the single transluminal gateway transcystic multiple drainages (SGTMD) procedure^6^ have been reported as additional endoscopic drainage techniques for multilocular WON with complex morphology. The main cavity and sub‐cavities basically communicate via narrow channels, and in SGTMD, these channels are probed from the main cavity with an endoscopic retrograde cholangiopancreatography catheter and guidewire, after which the sub‐cavities are drained by means of the placement of a double pigtail plastic stent or transnasal drainage tube. According to one study, in 93% of cases, WON can be cured by endoscopic treatment alone by means of MTGT or SGTMD.^7^ However, the maximum length of a double pigtail plastic biliary stent that can be used in Japan is 18 cm, so it is difficult to drain the sub‐cavity that exists in a location far from the digestive tract. Therefore, in this case, a double pigtail plastic ureteral stent was used. The maximum length of a double pigtail plastic ureteral stent is 30 cm, so the sub‐cavity of the pelvic cavity could be approached using it. However, it is not covered by insurance if it is used for anything other than the ureter, so it is necessary to obtain approval from the hospital ethics committee before using it. If large sub‐cavities have formed, the large amount of necrotic tissue will impede adequate drainage, and necrosectomy may be required. Nevertheless, inserting an endoscope into every corner of WON cavities with complex morphologies is technically challenging. If it is difficult to treat WON extending into the pelvic cavity by endoscopy alone, the additional use of percutaneous drainage has been reported to improve treatment results,^8^ and this should be actively considered as a supplement to endoscopic treatment. A hybrid approach to endoscopic treatment has also been reported that involves the concomitant use of video‐assisted retroperitoneal debridement, a minimally invasive surgical procedure.^9^ Endoscopic treatment is an excellent minimally invasive treatment method that maintains the patient's quality of life, but when endoscopic treatment is difficult, it is necessary to adopt a comprehensive strategy including a percutaneous approach and a surgical approach. In the present patient, the use of a LAMS enabled necrosectomy to be conducted multiple times in a stable manner, and the additional use of a double pigtail plastic ureteral stent enabled the successful improvement of WON in the pelvic cavity by endoscopic treatment alone.

## CONFLICT OF INTEREST

The authors declare no conflict of interest.

## FUNDING INFORMATION

None.

## ETHICS STATEMENT

All procedures were performed according to the principles of the Declaration of Helsinki and its later amendments.

## INFORMED CONSENT

Since it was difficult to obtain informed consent from the patient or his family, the ethics committee of our hospital approved the publication of the case details.

## Data Availability

All data generated or analyzed during this study are included in this published article.
